# Sex Hormone Receptors in Benign and Malignant Salivary Gland Tumors: Prognostic and Predictive Role

**DOI:** 10.3390/ijms19020399

**Published:** 2018-01-30

**Authors:** Gabriella Aquino, Francesca Collina, Rocco Sabatino, Margherita Cerrone, Francesco Longo, Franco Ionna, Nunzia Simona Losito, Rossella De Cecio, Monica Cantile, Giuseppe Pannone, Gerardo Botti

**Affiliations:** 1 Pathology Unit, Istituto Nazionale per lo Studio e la Cura dei Tumori, “Fondazione G. Pascale”, IRCCS, Naples 80131, Italy; gabryaquino@gmail.com (G.A.); francescacollina84@gmail.com (F.C.); roc.sabatino@gmail.com (R.S.); mcerrone1@virgilio.it (M.C.); s.losito@istitutotumori.na.it (N.S.L.); r.dececio@istitutotumori.na.it (R.D.C.); g.botti@istitutotumori.na.it (G.B.); 2 Head and Neck Surgery Unit, Istituto Nazionale per lo Studio e la Cura dei Tumori, “Fondazione G. Pascale”, IRCCS, Naples, 80131, Italy; f.longo@istitutotumori.na.it (F.L.); f.ionna@istitutotumori.na.it (F.I.); 3 Department of Clinical and Experimental Medicine, Pathological Anatomy Unit—University of Foggia, Foggia 71100, Italy; giuseppepannone@virgilio.it

**Keywords:** sex hormone receptors, salivary gland tumors, therapeutic targets

## Abstract

The role of sex hormone receptors in human cancer development and progression has been well documented in numerous studies, as has the success of sex hormone antagonists in the biological therapy of many human tumors. In salivary gland tumors (SGTs), little and conflicting information about the role of the estrogen receptor alpha (ERα), progesterone receptor (PgR) and androgen receptor (AR) has been described and in most cases the use of sex hormone antagonists is not contemplated in clinical practice. In this study, we analyzed a panel of sex hormone receptors that have not been widely investigated in SGTs—ERα, PgR, AR, but also ERβ and GPR30—to define their expression pattern and their prognostic and predictive value in a case series of 69 benign and malignant SGTs. We showed the aberrant expression of AR in mucoepidermoid and oncocytic carcinoma, a strong relation between cytoplasmic ERβ expression and tumor grade, and a strong correlation between nuclear GPR30 expression and disease-free survival (DFS) of SGT patients.

## 1. Introduction

Salivary gland tumors (SGT) are rare tumors, representing approximately 0.5% of all human cancers and less than 5% of head and neck lesions [1]. The WHO classification identifies 24 different malignant subtypes with different clinical courses and variable prognoses, mainly represented by primary epithelial tumors that account for approximately 88% of the SGTs [2]. Mucoepidermoid tumor (MEC), Salivary Duct Carcinoma (SDC) and adenoid cystic carcinoma (AdCC) represent the most frequent and often the more aggressive lesions [1]. Until today, surgical excision represents the only choice of treatment, with radio and/or chemotherapy in case of advanced disease and loco-regional recurrences. The application of new therapeutic strategies that are mainly based on the employment of biological drugs should be integrated into the management of these patients.

The overexpression of several sex hormone receptors, in particular, estrogen receptor alpha (ERα), progesterone receptor (PgR), and androgen receptor (AR), suggests their fundamental role in tumor pathogenesis and progression [3,4,5,6,7]. The production of sex hormone antagonists and their success in the treatment of patients with ERα+ and PgR+ breast carcinomas and AR+ prostate carcinomas have also suggested the investigation of the expression of these receptors in other tumors including SGTs [8]. 

Sex steroid hormones appear to play the main role in the physiology of the human oral cavity and salivary glands. However, most of the studies focused on the expression of ERα and PR report conflicting results. Alternatively, the expression and role of AR in SGTs are well documented [9,10]. The other estrogen receptor (ERβ) was described in salivary gland adenocarcinoma cell lines and certain salivary gland carcinomas such as AdCC and Pleomorphic adenoma (PA) [11,12]. The structure of ERβ is homologous to that of ERα and its DNA-binding domain is 96% conserved compared to ERα, suggesting that ERβ could bind the same target genes [13]. Specific ERβ isoforms are able to activate specific signal transduction pathways starting from the cytoplasm or plasma membrane, which may explain the effect of E2 in the modulation of cytoskeletal remodeling and the migration of salivary gland adenocarcinoma cells [14]. 

Whereas ERα and ERβ mediate the genomic estrogen signaling, the third membrane-bound Estrogen Receptor GPR30 (GPER) mediates the non-genomic signaling mechanisms. Several studies reported that the ligand activation of GPR30 signaling, coupled with the upregulation of specific GPER genes, was involved in the proliferation of tumor cells, suggesting that GPER can contribute to tumorigenesis [15,16]. On the role of GPR30 in SGTs, only one study showed GPR30 expression in oral epithelia like salivary glands and tongue [17].

Overall, little information is reported in the literature on the role of ERβ and GPR30 in SGTs. In this study, we aimed to analyze a panel of sex hormone receptors, such as ERα, ERβ, GPR30, PgR, and AR, in a case series of 75 SGTs of different histotypes to better define their expression pattern and their prognostic and predictive value in these tumors.

## 2. Results

### 2.1. Characteristics of SGTs Patients

In the study, only the patients with a complete panel of clinical-pathological features have been included, while Kaplan–Meier analysis has been carried out on selected patients with clinical outcome. The patients initially selected were 69 in number, and their samples have been included in Tissue Micro Array (TMA), however, the number of samples evaluable for statistical elaboration ranges from 54 to 62 cases, because of skipping cores for the different markers. The SGTs TMA was built with 36 cases of benign tumors (pleomorphic adenoma (PA), myoepithelioma, basal cell adenoma, Warthin tumor, and oncocytoma) and 33 cases of malignant tumors (MEC, acinic cell carcinoma (ACC), adenocarcinoma, mixed tumor, carcinoma ex pleomorphic adenoma (Ca ex PA), AdCC, oncocytic carcinoma and salivary duct carcinoma (SDC)). The prevalent location of these lesions is the parotid gland. All clinical pathological information of patients is summarized in Table 1.

### 2.2. Immunohistochemical Expression of AR, ERβ and GPR30, and Relation with Clinical-Pathological Features and Survival in SGTs

Little and often conflicting information about the role of sex hormone receptors in SGTs has been provided and, consequently, the use of specific biological drugs is not usually planned for these tumor diseases. For this reason, we analyzed a panel of sex hormone receptors in a case series of patients with benign and malignant SGTs. 

For all biomarkers, we considered both nuclear and cytoplasmic staining. Receptors ERα and PgR are never expressed in our series, in line with the literature [18]. In detail, we detected only nuclear AR expression in 15/61 (24%) of SGT samples in both malignant and benign SGT lesions. A total of eight cases were not considered evaluable.

**Figure 1:**
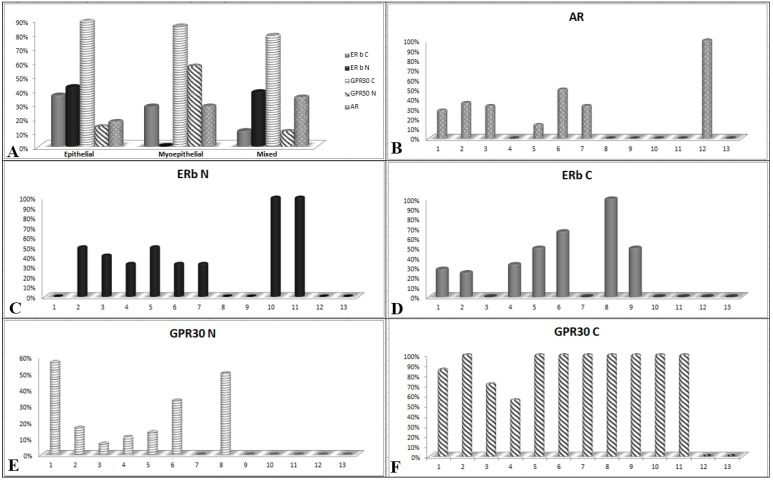
Schematic representation of distribution of Androgen Receptor (AR), Estrogen Receptor Beta (ERβ) and G protein-coupled receptor 30 (GPR30 IHC) expression in salivary gland tumors (SGTs): (**A**) AR, ERβ and GPR30 expression in cell differentiation SGT types (epithelial, myoepithelial and mixed); (**B**) nuclear AR expression in different histotypes; (**C**) nuclear ERβ expression in different SGT histotypes; (**D**) cytoplasmic ERβ expression in different SGT histotypes; (**E**) nuclear GPR30 expression in different SGT histotypes; (**F**) cytoplasmic GPR30 expression in different SGT histotypes. *X* = SGTs histotypes; *Y* = number of positive samples in percentage terms.

Considering the stratification of the lesions based on their cell differentiation, we detected AR expression in 17% of epithelial SGTs, in 28% of myoepithelial lesions and in 35% of mixed SGTs. (Figure 1A).

**Figure 2:**
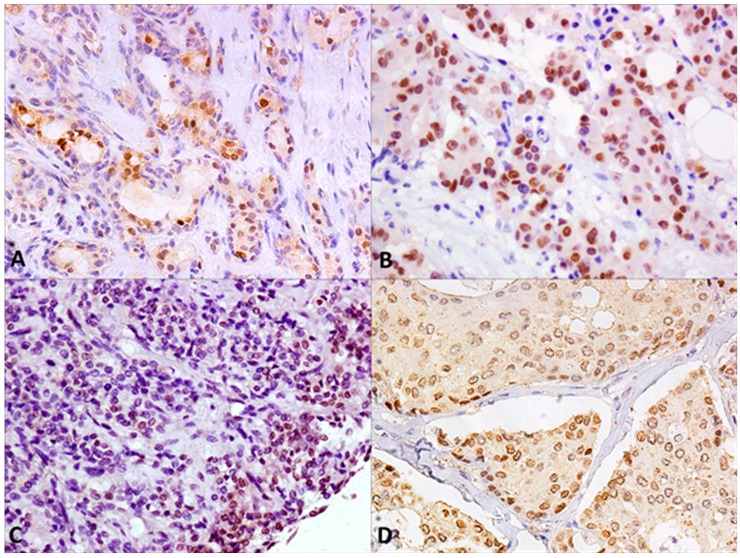
Nuclear AR IHC staining of SGTs samples: (**A**) Pleomorphic Adenoma (PA); (**B**) Oncocytic carcinoma; (**C**) Myoepithelioma; (**D**) Mucoepidermoid Carcinoma (MEC) (Magnification 20×).

In the context of benign lesions, AR was mainly expressed in PA (33%) and in 28% of myoepithelioma samples, and interestingly in 36% of MEC, in sporadic cases of Ca ex PA, and oncocytic carcinoma (Figure 1B and Figure 2). Their aberrant expression in malignant SGTs was sporadically reported in MEC, a very poor prognosis tumor, and never reported for oncocytic carcinoma, suggesting the use of AR antagonists in therapeutic strategies for these patients. 

Regarding ERβ we detected nuclear expression in 21/58 (36%) of SGTs and cytoplasmic staining in 16/58 (27%) of SGTs. We never detected nuclear and cytoplasmic ERβ co-expression. A total of 11 cases were not considered evaluable.

**Figure 3:**
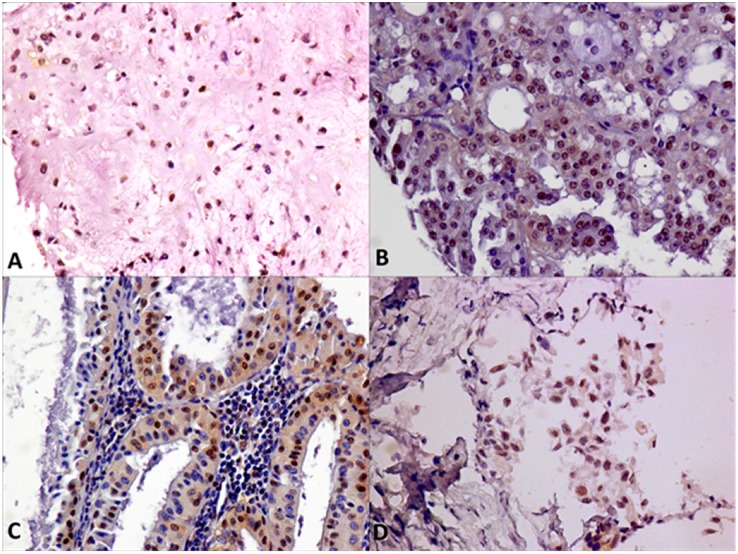
Nuclear ERβ IHC staining of SGTs samples: (**A**) PA; (**B**) acinic cell carcinoma (ACC); (**C**) Warthin’s tumor; (**D**) MEC (Magnification 20×).

**Figure 4:**
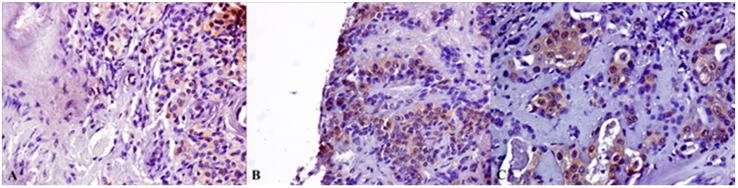
Cytoplasmic ERβ IHC staining of SGTs samples: (**A**) ACC; (**B**) Myoepithelioma; (**C**) MEC (Magnification 20×).

Regarding cell differentiation types, we detected cytoplasmic expression of ERβ in 36% of epithelial SGTs, in 28% of myoepithelial lesions, and in 11% of mixed SGTs. Nuclear expression was present in 42% epithelial SGTs, in 38% of mixed SGTs, and never detected in myoepithelial lesions (Figure 1A). In detail, nuclear ERβ was present in 40% of malignant lesions, mainly in 50% of MEC samples and in 33% of ACC. Moreover, we detected ERβ nuclear expression in 25% of benign lesions, mainly represented by PA and Warthin tumor (Figure 1C and Figure 3). Cytoplasmic expression of ERβ was detected in 33% of malignant lesions, mainly in 25% of MEC samples and in 33% of ACC, followed by sporadic cases of mixed tumors and adenocarcinoma. Moreover, we detected ERβ cytoplasmic expression in 19% of benign lesions, above all in myoepithelioma and Warthin tumors (Figure 1D and Figure 4). Also, in this case, the overexpression of ERβ in several malignant SGTs can suggest the use of antagonists of estrogen receptors, with equivalent affinities for ERβ and ERα [19], in these tumor patients.

Finally, we detected cytoplasmic staining of GPR30 in 34/62 (86%) of specimens with nuclear co-expression in 11/62 (18%) of SGTs. A total of 7 cases were not considered evaluable.

Regarding cell differentiation types we detected cytoplasmic expression of GPR30 in 88% of epithelial SGTs, in 85% of myoepithelial lesions and in 78% of mixed SGTs. Nuclear staining was detected respectively in 13%, 57% and 10% of epithelial, myoepithelial and mixed SGTs (Figure 1A).

**Figure 5:**
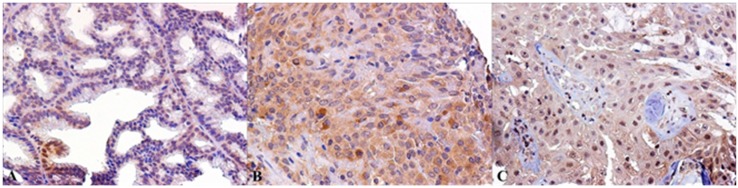
Nuclear GPR30 IHC staining of SGTs samples: (**A**) ACC; (**B**) Myoepithelioma; (**C**) MEC (Magnification 20×).

Cytoplasmic GPR30 expression was present in all cases of MEC and in most of other malignant lesions. In benign SGTs its expression was prevalent in myoepithelioma and PA samples. (Figure 5). 

**Figure 6:**
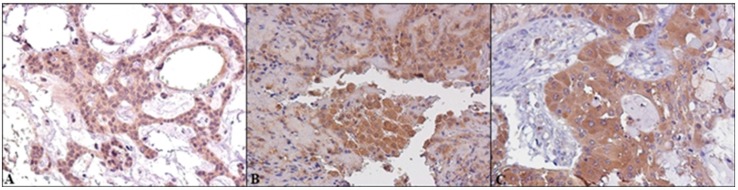
Cytoplasmic GPR30 IHC staining of SGTs samples: (**A**) PA; (**B**) Myoepithelioma; (**C**) MEC (Magnification 20×).

The nuclear GPR30 positivity was detected in 57% of myoepithelioma and in sporadic cases of PA and Warthin’s tumors. In malignant SGTs we detected nuclear GPR30 in 16% of MECs and in sporadic cases of ACCs, and adenocarcinoma (Figure 6). 

For the statistical elaboration, we considered tumor grade only in malignant tumors. Based on statistical elaboration of nuclear AR expression with the clinical-pathological features of SGTs, we showed no statistical significance with age, gender, site location, grade, cell differentiation, and proliferation index (Table 2). Cytoplasmic ERβ expression was significantly associated only with tumor grade (*p*-value = 0.052), while no statistical association with clinical-pathological features exist for nuclear ERβ expression. Similarly, no statistical association with clinical-pathological characteristics exist for cytoplasmic and nuclear GPR30 expression, except a trend of statistical association between cytoplasmic GPR30 expression and tumor grade (*p*-value = 0.087). All data are schematized in Table 2. 

**Figure 7:**
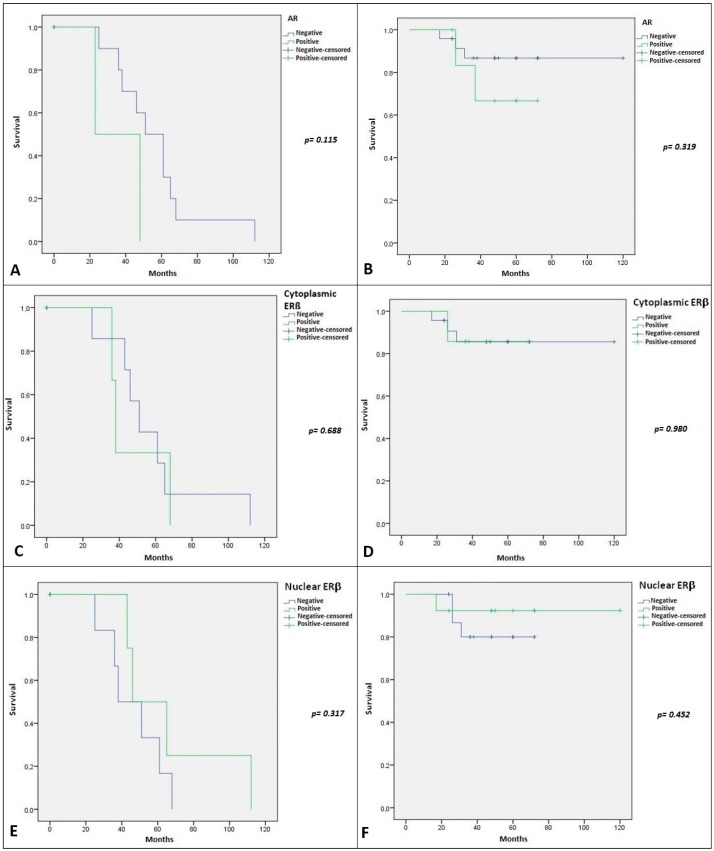
(**A**)Kaplan–Meier plot for disease-free survival (DFS) in patients with SGT stratified by AR IHC expression. The green line represents patients with AR nuclear positivity; (**B**) Kaplan–Meier plot for Overall survival (OS) in patients with SGT stratified by AR IHC expression. The green line represents patients with AR nuclear positivity; (**C**) Kaplan–Meier plot for DFS in patients with SGT stratified by cytoplasmic ERβ IHC expression. The green line represents patients with cytoplasmic ERβ positivity; (**D**) Kaplan–Meier plot for OS in patients with SGT stratified by Cytoplasmic ERβ IHC expression. The green line represents patients with cytoplasmic ERβ positivity; (**E**) Kaplan–Meier plot for DFS in patients with SGT stratified by nuclear ERβ IHC expression. The green line represents patients with nuclear ERβ positivity; (**F**) Kaplan–Meier plot for OS in patients with SGT stratified by nuclear ERβ *IHC* expression level. The green line represents patients with nuclear ERβ positivity.

**Figure 8:**
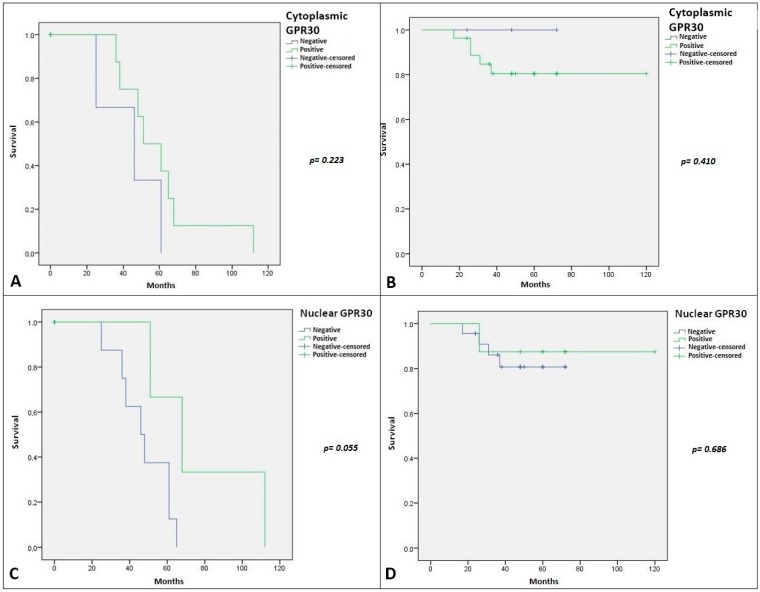
(**A**) Kaplan–Meier plot for disease-free survival (DFS) in patients with SGT stratified by Cytoplasmic GPR30 IHC expression. The green line represents patients with Cytoplasmic GPR30 positivity; (**B**) Kaplan–Meier plot for overall survival (OS) in patients with SGT stratified by GPR30 IHC expression. The green line represents patients with Cytoplasmic GPR30 positivity; (**C**) Kaplan–Meier plot for DFS in patients with SGT stratified by Nuclear GPR30 IHC expression (*p*-value = 0.055). The green line represents patients with Nuclear GPR30positivity; (**D**) Kaplan–Meier plot for OS in patients with SGT stratified by Nuclear GPR30 IHC expression. The green line represents patients with Nuclear GPR30 positivity.

Regarding the relation with clinical outcome of SGT patients, Kaplan–Meier curves referred to DFS and OS are illustrated in Figure 7 and Figure 8. We showed no statistical association with DFS and OS for both AR and nuclear and cytoplasmic expression of ERβ. Regarding GPR30 we showed a strong statistical significance between its nuclear expression and DFS (*p*-value = 0.055) (Figure 8D). The relationship between nuclear GPR30 and DFS highlighted the never reported prognostic role of this marker in SGTs.

## 3. Discussion

In recent years, many studies have focused on the expression of sex hormone receptors in human cancer and on the mechanisms through exerting their actions and influence the progression of tumor diseases. Moreover, the development of sex hormone antagonists and their successful employment in biological therapies for several tumors has suggested the evaluation of their expression and/or activity in different cancer types. However, in SGTs there is fragmentary and often conflicting information about the role of sex hormone receptors, and, for this reason, the use of biological drugs is not contemplated in clinical practice in the majority of the cases.

In our study, we analyzed a case series of patients with benign and malignant SGTs included in a TMA and correlated their expression with clinical-pathological parameters and outcomes.

In our SGT case series, we have never detected the expression of ERα and PgR. In literature, whereas benign salivary glands tumors were negative for hormone receptors expression [20], widely disparate results about ERα and PR expression in various malignant SGTs have been reported. Early studies showed immuno-positivity of ERα in 8% of SDC, with a total absence of PgR expression [21], a marked expression of PgR and absence of ERα expression in AdCC [22,23], while sporadic cases of ACC and MEC showed a positivity for both receptors [24]. Another study has described the absence of ERα expression both in AdCC and MEC [25]. More recent studies described ER and PgR positivity in only a few cases [20] while, as in our case, a large case series (139 salivary glands tumors) study never detected ERα and PgR positivity [18]. 

Regarding AR expression, we detected nuclear AR expression mainly in several benign lesions such as PA and myoepithelioma, but the aberrant AR expression was also identified in several malignant lesions. Whereas AR expression was abundantly documented in Ca ex PA, our data also showed the interesting expression in many cases of MEC and oncocytic carcinoma. 

A rich literature documented the expression and the role of AR in salivary glands tumors. Early studies described a very high IHC AR expression in SDC [9,10] with a more significant expression in men (79%) than in women (33%) [26]. Little information is available in literature about the role of AR in other SGTs. The absence of its expression was reported in AdCC, MEC, and ACC [27]. In PA a focal immunohistochemical expression of AR was described [28], while its expression was detected in 90% of Ca ex PA, suggesting an AR role in malignant tumor evolution [29].

Concerning the therapeutic potential of anti-AR drugs, several studies reported the benefits of anti-androgen therapy, in particular in the SDC histotype. In a series of 10 patients with an overexpression of AR, 50% of them was enormously benefited from treatment with bicalutamide [30]. Our data, in particular the aberrant expression of AR in several MEC and oncocytic carcinoma could suggest the potential use of anti-AR drugs also in these tumor types.

Regarding ERβ expression, we detected its positivity both at nuclear and cytoplasmic level with a prevalent expression in epithelial malignant lesions such as MEC samples and ACC, while myoepithelial lesions never present ERβ nuclear expression.

Expression of ERβ was reported at high levels in oral tissues, mainly in keratinocytes and salivary gland acinar and ductal cells [31]. Overexpression of ER-β was described in four cases of pediatric MEC and in ACC cell line [32], while nuclear overexpression of ER-β was detected also in 71% of ACC FPPE tissues, with the average expressions higher in women, and in the cases with a cribriform architecture [11]. ERβ was also detected in several cases of PA of the salivary gland [12]. 

Several studies showed that antagonists of estrogen receptors, can have therapeutic effects in preclinical models, in particular in ERβ+ TNBC models. Oral estradiol, approved for treatment of metastatic breast cancer has equivalent affinities for ERβ and ERα [19]. In fact, ERβ can bind other ligands with rather higher affinity than ERα, such as 4-hydroxytamoxifen, the phytoestrogen genistein, and, testosterone derivatives, 3βAdiol [33]. This suggests the possibility of its use to target ERβ in TNBC [29] but also in other ERα+ tumors. Moreover, several studies showed that higher ERβ expression was an independent predictor of better tamoxifen response [34,35] and overexpression of ERβ1 was also associated with increased sensitivity to 4-hydroxytamoxifen [36].

In our SGTs case series, while the nuclear ERβ expression does not appear to be associated with clinical outcomes, cytoplasmic ERβ staining showed a strong association with tumor grade, highlighting its strong prognostic value. It was reported that different ERβ variant isoforms can be localized in the cytoplasm and plasma membrane, showing variable expression in cancer tissues and influencing cancer progression and response to therapy [37]. Our results suggest that cytoplasmic ERβ signaling in SGTs may be more important for patient outcome than its nuclear signaling. This is probably due to ERβ2 isoform which is already documented to be strongly related to poorer prognosis in breast cancer [38]. Several studies showed the same findings in other tumor types, such as ovarian cancer, squamous cell carcinoma [39,40]. For these tumors, the use of estrogen receptor antagonists could be suggested in clinical practice. 

Only one study in the literature reported the expression of GPR30 in oral tissues [20]. GPR30 (GPER), as a 7-transmembrane GPCR and is predominantly, though not exclusively, localized on intracellular membranes, particularly on those of the endoplasmic reticulum and Golgi apparatus [41] in several tissues such as reproductive tissues, heart, intestines, ovary, CNS, pancreatic islets, adipose tissue, skeletal muscle, liver, neurons, and inflammatory cells [42]. 

We detected its cytoplasmic staining in most SGT specimens, particularly in MEC. Furthermore, its nuclear staining was prevalent in several benign lesions but also in a discrete number of MEC and ACC. Cheng et al. demonstrated that retrograde transport of GPR30 from the plasma membrane towards the nucleus occurs with a consecutive accumulation of GPR30 in the perinuclear space followed by a later dispersion in the cytoplasm [43]. Recent studies showed that the different location of GPR30—cytoplasmic and nuclear locations—can reflect distinct tumor properties in breast cancer [44], and the lack of GPR30 expression in the plasma membrane can be associated with excellent long-term prognosis in ERα and PgR-positive tamoxifen-treated primary breast cancer [45]. This trend reflects our data. In fact, in our series nuclear expression of GPR30, it was statistically associated with a better DFS in SGT patients. Although the subcellular GPR30 trafficking process (which is probably related to a functional receptor modulation) has never been described in SGTs, we can speculate a dynamic intracellular shift strongly related to SGT cancer progression. 

A non-steroidal, high-affinity GPR30 agonist G-1 has been developed to dissect GPR30-mediated estrogen responses from those mediated by classic estrogen receptors [46]. Moreover, several highly selective GPR30 antagonists, such as G15 and G36, were identified [47]. In particular, G36 has a better activity compared to G15 in a range of functional assays, both in vitro and in vivo [48]. In an endometrial tumor cell model, G36 greatly reduces growth of estrogen-stimulated cells, suggesting that GPR30 may play a critical role in endometrial carcinogenesis and, therefore, providing G36 as a novel target for prognosis and treatment [49]. 

In conclusion, our data highlighted the aberrant expression of several sex hormone receptors, in particular of alternative estrogen receptors, such as ERβ and GPR30 in SGTs, showing their prognostic value and suggesting consideration of them as new biological targets.

## 4. Material and Methods

### 4.1. Patients with Salivary Glands Tumors

75 patients admitted to the National Cancer Institute “Giovanni Pascale” of Naples, between 2012 and 2017, were recruited in this study. All patients had provided written informed consent for the use of samples according to the institutional regulations and the study was approved by the ethics committee of the National Cancer Institute “Giovanni Pascale” and was registered “Bio-Banca Istituzionale BBI” Deliberation (NO. 15 del, 20 Jan. 2016).

All cases have been reviewed according to WHO 2017 classification criteria [2] using standard tissue sections. Clinic-pathological characteristics, including tumor–node–metastasis (TNM) stage, were collected. Medical records have been reviewed for clinical information, including histologic parameters assessed on standard H&E-stained slides.

### 4.2. TMA Building

A Prognostic-Tumor Array was built using 75 tumor tissue samples. H&E staining of 4 μm TMA section was used to verify all samples. One core from tumor areas of each subtype tumor was arrayed in a recipient block. All tumors and controls were reviewed by two experienced pathologists (Giuseppe Pannone and Nunzia Simona Losito). Discrepancies for the same case were resolved in a joint analysis. Tissue cylinders with a diameter of 1 mm were punched from morphologically representative tissue areas of each “donor” tissue block and brought into one recipient paraffin block (3 core of tissue × 1 mm) using a semi-automated tissue array (Galileo TMA CK 3500 Tissue Micro arrayer; ISE TMA Software, Integrated System Engineering, Milano, Italy).

### 4.3. Immunohistochemistry Analysis

Immunohistochemical staining was carried out on slides from formalin-fixed, paraffin embedded tissues (FPPE), in order to evaluate the expression of ERα, ERβ, GPR30, PgR, and AR. FPPE slides were de-paraffinized in xylene and rehydrated through graded alcohols. Antigen retrieval was performed with slides heated in 0.0.1 M citrate buffer (pH 6.0) in a bath for 20 min at 97 °C. After antigen retrieval, the slides were allowed to cool. The slides were rinsed with TBS and the endogenous peroxidase has inactivated with 3% hydrogen peroxide. After protein block (BSA 5% in PBS 1x), the slides were incubated with primary antibody to human ERα (Monoclonal Mouse Anti-Human ERα, Clone ID5, dilution 1:35, Dako North America, Inc., Carpinteria, CA, USA), PR (Monoclonal Mouse Anti-Human PR, Clone 636, dilution 1:50, Dako North America, Inc., Carpinteria, CA, USA), Ki67 (Monoclonal Mouse Anti-Human Ki67 Ag Clone MIB-1, dilution 1:75, Dako North America, Inc., Carpinteria, CA, USA) for 30 min, AR (monoclonal mouse anti-human AR antibody clone AR441, dilution 1:75, #M3562; Dako North America, Inc., Carpinteria, CA, USA), GPR30 (polyclonal rabbit antibody, clone sc-48524-R, dilution 1:300, Santa Cruz Biotechnology, Dallas, TX, USA) and ERβ (Monoclonal Mouse Anti-Human ERβ, clone PPG5/10, dilution 1:30, Dako North America, Inc., Carpinteria, CA, USA) overnight. Sections were incubated with mouse anti-rabbit or goat anti-mouse secondary IgG biotinylated secondary antibody for 30 min. Immunoreactivity was visualized by means of avidin–biotin–peroxidase complex kit reagents (Novocastra, Newcastle, UK) as the chromogenic substrate. Finally, sections were weakly counterstained with hematoxylin and mounted.

### 4.4. Evaluation of Immunostaining

Antigen expression was independently evaluated by two experienced pathologists (GP/SL) using light microscopy. All values of immunostaining were expressed in percentage terms of positive cells and intensity. The percentage of positive cancer cells was evaluated in each sample by counting the number of positive cells over the total cancer cells in 10 non-overlapping fields using 400× magnification. The cutoff used to distinguish “positive” from “negative” cases was ≥1% ERα/PR positive tumor cells. For the proliferative index Ki67 was defined as the percentage of immuno-reactive tumor cells out of the total number of cells. The percentage of positive cells per case was scored according to 2 different groups: group 1: <5% (low proliferative activity); group 2: >5% (high proliferative activity). For nuclear AR expression the cutoff used to distinguish “positive” from “negative” cases was ≥1% AR-positive tumor cells. For ERβ expression was considered the percentage of positive cells for both nuclear and cytoplasmic staining. For GPR30, being positive in the most of cells for each sample, we considered the intensity of the reaction as negative, weak, intermediate, and strong (0, 1+, 2+, 3+) (Supplementary Table S1). 

### 4.5. Statistical Analysis

The association between ERα, ERβ, GPR30, PgR and AR expression with clinical-pathological parameters and was conducted using the χ^2^ and Student’s *t*-test.

The Pearson χ^2^ test was used to determine whether a relationship existed between the variables included in the study. The level of significance was defined as *p* < 0.05. Overall survival (OS) and disease-free survival (DFS) curves were calculated using the Kaplan–Meier method with significance valuated using the Mantel–Cox log-rank test. All the statistical analyses were carried out using the Statistical Package for Social Science v. 20 software (SPSS Inc., Chicago, IL, USA). OS was defined as the time from diagnosis (first biopsy) to death by any cause or until the most recent follow-up. DFS was measured as the time from diagnosis to the occurrence of progression, relapse after complete remission, or death from any cause. DFS had a value of zero for patients who did not achieve complete remission. The follow-up duration was five years.

## Figures and Tables

**Table 1:** Main Clinical-Pathological data.

Patient Features	Number of Patients	69
	Median Age (Range)	60 (17–87) Years
**Sex**	Male	41 (59.4%)
	Female	28 (40.6%)
**Lesion**	Benign	36 (52.2%)
	Malign	33 (47.8%)
**Site**	Parotid	59 (85.5%)
	SG	10 (14.5%)
**Grading**	G1	14 (42.4%)
	G2/G3	19 (57.6%)
	Benign (without grading)	36
**Ki67 Score**	≤5%	42 (60.9%)
	>5%	20 (29%)
	NA	7 (10.1%)
**Cell Type Differentiation**	Epithelial	38 (55.1%)
	Myoepithelial	7 (10.1%)
	Mixed	24 (34.8%)
**Histotype**	MEC	13 (18.8%)
	ACC	9 (13%)
	CA ex PA	3 (4.3%)
	Adenocarcinoma	2 (2.9%)
	AdCC	1 (1.4%)
	SDC	1 (1.4%)
	Oncocytic CA	1 (1.4%)
	Mixed tumor	3 (4.3%)
	PA	18 (26.1%)
	Warthin’s tumors	9 (13%)
	Myoepithelioma	7 (10.1%)
	Oncocytoma	1 (1.4%)
	Basal cell adenoma	1 (1.4%)

SG: Salivary Galnd; G1: Grading 1 G2: Grading 2 G3: Grading 3; MEC: Mucoepidermoid carcinoma; ACC: Acinic cell carcinoma; CA: Carcinoma; PA: Pleomorphic adenoma; AdCC: Adenoid cystic carcinoma SDC: Salivary ductal carcinoma.

**Table 2:** Statistical association of AR, ERβ and GPR30 tumor expression with clinical pathological features of SGT patients. (SG = Submandibular Gland)

Patient Features		Nuclear AR				Cytoplasmic ERβ				Nuclear ERβ				Cytoplasmic GPR30				Nuclear GPR30			
		Negative	Positive	*p*-Value	R Pearson	Negative	Positive	*p*-Value	R Pearson	Negative	Positive	*p*-Value	R Pearson	Negative	Positive	*p* Value	R Pearson	Negative	Positive	*p*-Value	R Pearson
Age	≤60	24	10	0.326	−0.126	25	6	0.133	0.197	18	13	0.331	−0.128	3	29	0.235	−0.151	24	8	0.122	−0.196
	>60	22	5			17	10			19	8			6	24			27	3		
Sex	Male	27	8	0.715	0.047	25	10	0.836	−0.027	22	13	0.855	−0.024	3	32	0.130	−0.192	30	5	0.417	0.103
	Female	19	7			17	6			15	8			6	21			21	6		
Site	Parotid	41	13	0.795	0.033	35	14	0.695	−0.051	32	17	0.576	0.073	8	45	0.754	0.040	42	11	0.132	−0.191
	SG	5	2			7	2			5	4			1	8			9	0		
Lesion	Benign	22	9	0.413	−0.105	21	6	0.394	0.112	18	9	0.671	0.056	4	27	0.718	−0.046	24	7	0.319	−0.127
	Malignant	24	6			21	10			19	12			5	26			27	4		
Grade	G1	10	2	0.709	0.068	12	2	0.052	0.349	9	5	0.756	0.056	4	10	0.087	0.307	13	1	0.385	0.156
	G2–G3	14	4			9	8			10	7			1	16			14	3		
Ki67	≤5%	29	9	0.904	−0.016	26	9	0.392	0.116	23	12	0.570	0.077	8	31	0.150	0.191	32	7	0.704	0.050
	>5%	14	4			12	7			11	8			1	17			14	4		

## Supplementary Materials

Supplementary materials can be found at http://www.mdpi.com/1422-0067/19/2/399/s1.

## References

[1] Cheu W., Chan J.K., Fletcher C.D. (2000). Salivary gland tumours. Diagnostic Histopathology of Tumours.

[2] El-Naggar A.K., Chan J.K.C., Grandis J.R., Takata T., Slootweg P.J. (2017). WHO Classification of Head and Neck Tumours.

[3] Rochefort H., Chalbos D. (2010). The role of sex steroid receptors on lipogenesis in breast and prostate carcinogenesis: A viewpoint. Horm. Cancer.

[4] Townsend E.A., Miller V.M., Prakash Y.S. (2012). Sex differences and sex steroids in lung health and disease. Endocr. Rev..

[5] González-Arenas A., Agramonte-Hevia J. (2012). Sex steroid hormone effects in normal and pathologic conditions in lung physiology. Mini Rev. Med. Chem..

[6] Cleve A., Fritzemeier K.H., Haendler B., Heinrich N., Möller C., Schwede M., Wintermantel T. (2012). Pharmacology and clinical use of sex steroid hormone receptor modulators. Handb. Exp. Pharmacol..

[7] Higa G.M., Fell R.G. (2013). Sex Hormone Receptor Repertoire in Breast Cancer. Int. J. Breast. Cancer.

[8] Giovannelli P., Di Donato M., Giraldi T., Auricchio F. (2011). Targeting rapid action of sex steroid receptors in breast and prostate cancers. Front Biosci..

[9] Kapadia S.B., Barnes L. (1998). Expression of androgen receptor, gross cystic disease fluid protein, and CD44 in salivary duct carcinoma. Mod. Pathol..

[10] Fan C.Y., Melhem M.F., Hosal A.S., Grandis J.R., Barnes E.L. (2001). Expression of androgen receptor, epidermal growth factor receptor, and transforming growth factor α in salivary duct carcinoma. Arch. Otolaryngol.-Head Neck Surg..

[11] Marques Y.M., Giudice F.S., Freitas V.M., Abreu e Lima Mdo C., Hunter K.D., Speight P.M., Machado de Sousa S.C. (2012). Oestrogen receptor β in adenoid cystic carcinoma of salivary glands. Histopathology.

[12] Wong M.H., Dobbins T.A., Tseung J., Tran N., Lee C.S., O’Brien C.J., Clark J., Rose B.R. (2009). Oestrogen receptor β expression in pleomorphic adenomas of the parotid gland. J. Clin. Pathol..

[13] Kuiper G.G., Enmark E., Pelto-Huikko M., Nilsson S., Gustafsson J.A. (1996). Cloning of a novel receptor expressed in rat prostate and ovary. Proc. Natl. Acad. Sci. USA.

[14] Ohshiro K., Rayala S.K., Williams M.D., Kumar R., El-Naggar A.K. (2006). Biological Role of Estrogen Receptor β in Salivary Gland Adenocarcinoma Cells. Clin. Cancer Res..

[15] Albanito L., Madeo A., Lappano R., Vivacqua A., Rago V., Carpino A., Oprea T.I., Prossnitz E.R., Musti A.M., Andò S., Maggiolini M. (2007). G protein-coupled receptor 30 (GPR30) mediates gene expression changes and growth response to 17β-estradiol and selective GPR30 ligand G-1 in ovarian cancer cells. Cancer Res..

[16] Lappano R., Santolla M.F., Pupo M., Sinicropi M.S., Caruso A., Rosano C., Maggiolini M. (2012). MIBE acts as antagonist ligand of both estrogen receptor α and GPER in breast cancer cells. Breast Cancer Res..

[17] Mau M., Mielenz M., Südekum K.H., Obukhov A.G. (2011). Expression of GPR30 and GPR43 in oral tissues: Deriving new hypotheses on the role of diet in animal physiology and the development of oral cancers. J. Anim. Physiol. Anim. Nutr..

[18] Locati L.D., Perrone F., Losa M., Mela M., Casieri P., Orsenigo M., Cortelazzi B., Negri T., Tamborini E., Quattrone P. (2009). Treatment relevant target immunophenotyping of 139 salivaryglandcarcinomas (SGCs). Oral Oncol..

[19] Kuhl H. (2005). Pharmacology of estrogens and progestogens: Influence of different routes of administration. Climacteric.

[20] Nasser S.M., Faquin W.C., Dayal Y. (2003). Expression of androgen, estrogen, and progesterone receptors in salivary gland tumors. Frequent expression of androgen receptor in a subset of malignant salivary gland tumors. Am. J. Clin. Pathol..

[21] Barnes L., Rao U., Contis L., Krause J., Schwartz A., Scalamogna P. (1994). Salivary duct carcinoma. Part II. Immunohistochemical evaluation of 13 cases for estrogen and progesterone receptors, cathepsin D, and C-ERBB-2 protein. Oral. Surg. Oral. Med. Oral. Pathol..

[22] Shick P.C., Riordan G.P., Foss R.D. (1995). Estrogen and progesterone receptors in salivary gland adenoid cystic carcinoma. Oral. Surg. Oral. Med. Oral. Pathol. Oral. Radiol. Endod..

[23] Dori S., Trougouboff P., David R., Buchner A. (2000). Immunohistochemical evaluation of estrogen and progesterone receptors in adenoid cystic carcinoma of salivary gland origin. Oral Oncol..

[24] Jeannon J.P., Soames J.V., Bell H., Wilson J.A. (1999). Immunohistochemical detection of oestrogen and progesterone receptors in salivary tumours. Clin. Otolaryngol. Allied Sci..

[25] Pires F.R., da Cruz Perez D.E., de Almeida O.P., Kowalski L.P. (2004). Estrogen receptor expression in salivary gland mucoepidermoid carcinoma and adenoid cystic carcinoma. Pathol. Oncol. Res..

[26] Williams M.D., Roberts D., Blumenschein G.R., Temam S., Kies M.S., Rosenthal D.I., Weber R.S., El-Naggar A.K. (2007). Differential expression of hormonal and growth factor receptors in salivary duct carcinomas: Biologic significance and potential role in therapeutic stratification of patients. Am. J. Surg. Pathol..

[27] Sygut D., Bień S., Ziółkowska M., Sporny S. (2008). Immunohistochemical expression of androgen receptor in salivary gland cancers. Pol. J. Pathol..

[28] Moriki T., Ueta S., Takahashi T., Mitani M., Ichien M. (2001). Salivary duct carcinoma: Cytologic characteristics and application of androgen receptor immunostaining for diagnosis. Cancer.

[29] Nakajima Y., Kishimoto T., Nagai Y., Yamada M., Iida Y., Okamoto Y., Ishida Y., Nakatani Y., Ichinose M. (2009). Expressions of androgen receptor and its co-regulators in carcinoma ex pleomorphic adenoma of salivary gland. Pathology.

[30] Jaspers H.C., Verbist B.M., Schoffelen R., Mattijssen V., Slootweg P.J., van der Graaf Winette T.A., Carla M.L., van Herpen C.M. (2011). Androgen receptor-positive salivary duct carcinoma: A disease entity with promising new treatment options. J. Clin. Oncol..

[31] Välimaa H., Savolainen S., Soukka T., Silvoniemi P., Mäkelä S., Kujari H., Gustafsson J.A., Laine M. (2004). Estrogen receptor-β is the predominant estrogen receptor subtype in human oral epithelium and salivary glands. J. Endocrinol..

[32] Locati L.D., Collini P., Imbimbo M., Barisella M., Testi A., Licitra L.F., Löning T., Tiemann K., Quattrone P., Bimbatti E. (2017). Immunohistochemical and molecular profile of salivary gland cancer in children. Pediatr. Blood Cancer.

[33] Omoto Y., Iwase H. (2015). Clinical significance of estrogen receptor β in breast and prostate cancer from biological aspects. Cancer Sci..

[34] Hopp T.A., Weiss H.L., Parra I.S., Cui Y., Osborne C.K., Fuqua S.A. (2004). Low levels of estrogen receptor β protein predict resistance to tamoxifen therapy in breast cancer. Clin. Cancer Res..

[35] Iwase H., Zhang Z., Omoto Y., Sugiura H., Yamashita H., Toyama T., Iwata H., Kobayashi S. (2003). Clinical significance of the expression of estrogen receptors alpha and β for endocrine therapy of breast cancer. Cancer Chemother. Pharmacol..

[36] Murphy L.C., Peng B., Lewis A., Davie J.R., Leygue E., Kemp A., Ung K., Vendetti M., Shiu S. (2005). Inducible upregulation of oestrogen receptor-β1 affects oestrogen and tamoxifen responsiveness in MCF7 human breast cancer cells. J. Mol. Endocrinol..

[37] Thomas C., Gustafsson J.Å. (2011). The different roles of ER subtypes in cancer biology and therapy. Nat Rev Cancer..

[38] Shaaban A.M., Green A.R., Karthik S., Alizadeh Y., Hughes T.A., Harkins L., Ellis I.O., Robertson J.F., Paish E.C., Saunders P.T., Groome N.P. (2008). Nuclear and cytoplasmic expression of ERβ1, ERβ2, and ERβ5 identifies distinct prognostic outcome for breast cancer patients. Clin Cancer Res..

[39] De Stefano I., Zannoni G.F., Prisco M.G., Fagotti A., Tortorella L., Vizzielli G., Mencaglia L., Scambia G., Gallo D. (2011). Cytoplasmic expression of estrogen receptor β (ERβ) predicts poor clinical outcome in advanced serous ovarian cancer. Gynecol Oncol..

[40] Zannoni G.F., Prisco M.G., Vellone V.G., De Stefano I., Vizzielli G., Tortorella L., Fagotti A., Scambia G., Gallo D. (2011). Cytoplasmic expression of oestrogen receptor β (ERβ) as a prognostic factor in vulvar squamous cell carcinoma in elderly women. Histopathology..

[41] Prossnitz E.R., Arterburn J.B., Sklar L.A. (2007). GPR30: AG protein-coupled receptor for estrogen. Mol. Cell. Endocrinol..

[42] Gaudet H.M., Cheng S.B., Christensen E.M., Filardo E.J. (2015). The G-protein coupled estrogen receptor, GPER: The inside and inside-out story. Mol. Cell. Endocrinol..

[43] Cheng S.B., Graeber C.T., Quinn J.A., Filardo E.J. (2011). Retrograde transport of the transmembrane estrogen receptor, G-protein-coupled-receptor-30 (GPR30/GPER) from the plasma membrane towards the nucleus. Steroids..

[44] Samartzis E.P., Noske A., Meisel A., Varga Z., Fink D., Imesch P. (2014). The G Protein-Coupled Estrogen Receptor (GPER) Is Expressed in Two Different Subcellular Localizations Reflecting Distinct Tumor Properties in Breast Cancer. PLoS ONE.

[45] Sjöström M., Hartman L., Grabau D., Fornander T., Malmström P., Nordenskjöld B., Sgroi D.C., Skoog L., Stål O., Leeb-Lundberg L.M. (2014). Lack of G protein-coupled estrogen receptor (GPER) in the plasma membrane is associated with excellent long-term prognosis in breast cancer. Breast Cancer Res. Treat..

[46] Bologa C.G., Revankar C.M., Young S.M., Edwards B.S., Arterburn J.B., Kiselyov A.S., Parker M.A., Tkachenko S.E., Savchuck N.P., Sklar L.A. (2006). Virtual and biomolecular screening converge on a selective agonist for GPR30. Nat. Chem. Biol..

[47] Dennis M.K., Burai R., Ramesh C., Petrie W.K., Alcon S.N., Nayak T.K., Bologa C.G., Leitao A., Brailoiu E., Deliu E. (2009). In vivo effects of a GPR30 antagonist. Nat. Chem. Biol..

[48] Dennis M.K., Field A.S., Burai R., Ramesh C., Petrie W.K., Bologa C.G., Oprea T.I., Yamaguchi Y., Hayashi S., Sklar L.A. (2011). Identification of a GPER/GPR30 Antagonist with Improved Estrogen Receptor Counter selectivity. J. Steroid Biochem. Mol. Biol..

[49] Petrie W.K., Dennis M.K., Hu C., Dai D., Arterburn J.B., Smith H.O., Hathaway H.J., Prossnitz E.R. (2013). G Protein-Coupled Estrogen Receptor-Selective Ligands Modulate Endometrial Tumor Growth. Obstet. Gynecol. Int..

